# Barriers in detecting elder abuse among emergency medical technicians

**DOI:** 10.1186/s12873-016-0100-7

**Published:** 2016-09-02

**Authors:** Jennifer M. Reingle Gonzalez, M. Brad Cannell, Katelyn K. Jetelina, Sepeadeh Radpour

**Affiliations:** 1 Department of Epidemiology, Human Genetics and Environmental Sciences, UT School of Public Health, Dallas Regional Campus, 6011 Harry Hines Blvd. V.8.112, Dallas, TX 75390 USA; 2 Department of Biostatistics and Epidemiology, University of North Texas Health Science Center, Fort Worth, TX USA

**Keywords:** Elder abuse, Neglect, Exploitation, Older adult, Screening

## Abstract

**Background:**

Elder abuse and neglect are highly under-reported in the United States. This may be partially attributed to low incidence of reporting among emergency medical technicians’ (EMTs), despite state-mandated reporting of suspected elder abuse. Innovative solutions are needed to address under-reporting. The objective was to describe EMTs’ experience detecting and reporting elder abuse.

**Methods:**

Qualitative data were collected from 11 EMTs and 12 Adult Protective Services (APS) caseworkers that participated in one of five semi-structured focus groups. Focus group data were iteratively coded by two coders.

**Results:**

Findings suggest a number of barriers prevent EMTs from reporting elder abuse to APS. Participants suggested that limited training on elder abuse detection or reporting has been provided to them. EMTs suggested that training, creation of an automated reporting system or brief screening tool could be used to enhance EMT’s ability to detect and communicate suspected cases of elder abuse to APS.

**Conclusions:**

Results from the present study suggest that EMTs may be uniquely situated to serve as elder abuse and neglect surveillance personnel. EMTs are eager to work with APS to address the under-reporting of elder abuse and neglect, but training is minimal and current reporting procedures are time-prohibitive given their primary role as emergency healthcare providers. Future studies should seek to translate these findings into practice by identifying specific indicators predictive of elder abuse and neglect for inclusion on an automated reporting instrument for EMTs.

**Electronic supplementary material:**

The online version of this article (doi:10.1186/s12873-016-0100-7) contains supplementary material, which is available to authorized users.

## Background

Community-dwelling older adults in the United States who experience abuse or neglect have approximately 200–400 % greater odds of mortality when compared to older adults who do not experience abuse or neglect [1, 2]. Chronic morbidities, including depression or anxiety, chronic pain, high blood pressure and heart problems, are also more common among adults over 65 who are not abused or neglected [2–5]. And although the true economic costs of Elder Abuse have yet to be quantified with certainty, estimates reach into the billions of dollars each year [6].

Despite these poor outcomes, elder abuse and neglect are highly under-reported in the United States (U.S.) [7–10]. Estimates suggest that between 1.6 and 11 % of older adults experience abuse each year [11], but only 1 in as many as 24 cases of elder abuse are reported to the authorities [7, 9, 12]. Similarly, using both APS records and self-report data, results from one of the most comprehensive epidemiological studies on the prevalence of elder abuse to date found that 76 out of every 1000 older adults in New York were confirmed victims of elder abuse between 2008 and 2009, and 141 of every 1000 older adults were victims of some form of elder abuse at least once since turning 60 [9]. Of these victims, only 3.24 per 1000 older adults contacted social services or law enforcement for investigation and support. [9] Most of these cases were neglect by a third party (e.g., not self-neglect) (64 %) or abuse (19 %) [9]. Therefore, it is clear that new strategies that enhance detection are necessary to reduce the burden of abuse and neglect among older adults.

The Fort Worth [TX] Safe Communities Coalition (FWSCC) is a member of Safe Communities America, an accreditation program of the National Safety Council. FWSCC includes stakeholders from local government and almost 200 other organizations, including Universities located in the Dallas/Fort Worth metropolitan area. FWSCC is made up of multiple task forces that seek to use collaborative, evidence-based methods for injury prevention and health promotion across the city [13]. During a meeting of the FWSCC Elder Abuse Task Force, the authors identified an unexpected trend in elder abuse reporting among emergency medical technicians (EMTs) employed by the largest mobile healthcare provider in North Texas. Data from this provider suggest that only 23 incidents of suspected elder abuse were reported to Adult Protective Services (APS) by EMTs in 2013, despite state-mandated reporting of suspected elder abuse, neglect or exploitation in Texas [14, 15]. During the same year, EMTs responded to more than 30,000 calls for emergency services for older adults. Given the prevalence rates described above, we would expect to observe 480–3000 cases of elder abuse and/or neglect that were not detected or reported [7, 12]. This is particularly concerning because social isolation, dementia, and health and functional status are risk factors for elder abuse [16], and may hinder older adults’ ability to self-identify and self-report abuse or neglectful situations [17]. Alternatively, older adults with cognitive or functional limitations may fear retaliation by a family member or caregiver, and as a result, continue to live in abusive or neglectful situations.

Because older adults are four-times more likely to use in-home emergency medical services than younger adults [18], EMTs are uniquely positioned to identify potential abusive or neglectful situations. EMTs can identify indicators of abuse or neglect (such as family interactions, home upkeep, medication availability, safety concerns and sanitation) not available to other emergency personnel, social workers or health care providers. In an effort to increase reporting and detection of abuse and neglect in North Texas, we conducted a qualitative investigation to understand how EMTs might act as surveillance personnel to enhance the detection, reporting, and investigation of suspected elder abuse and neglect cases. The long-term purpose of this project is to develop a screening tool for use by EMTs to streamline identification of potentially abusive or neglectful situations, reporting these cases to APS, and APS investigation. The first step in this process was to identify the primary barriers that currently exist to reporting suspected cases of elder abuse as perceived by EMTs, and to determine whether EMTs would be use a screening tool if it was available.

## Methods

### Setting

Details related to the study design and adherence to qualitative research protocols are described in Table 1 [19]. Participants were conveniently recruited from two agencies: 1) a large mobile healthcare provider in North Texas; and, 2) a regional APS office that serves the same metropolitan area. The mobile healthcare provider employs 220 EMTs that provide advanced life support to residents over a 15-county region (>100,000 service calls annually). The provider also offers innovative, preventive services to the community, including home visits for patient navigation among those who use the emergency department frequently, in-home care management visits, and home health partnerships. APS was included in this study because they are charged with investigating cases of elder abuse, neglect, and exploitation reported by parties that suspect abuse, including EMTs. Therefore, an assessment of EMT-APS communication and barriers to detection and reporting of abuse would be impossible without APS input.

**Table 1:** Consolidated criteria for reporting qualitative studies (COREQ) checklist [19]

	Investigators (*n* = 2)	Graduate research assistants (GRAs; *n* = 4)
Domain 1: Research team and reflexivity		
Personal characteristics		
Interview/facilitator	At least one Investigator led each of the 5 focus groups	Three of the four project GRAs assisted in focus group administration as note-takers
Credentials	PhD	1) A doctoral candidate with a MPH degree
		2) A medical student
		3) M.S. student with experience in qualitative research
		4) Recent MPH graduate
Occupation	Assistant Professors at large research universities in the Dallas-Fort Worth area	All GRAs were current students during the time of the study
Gender	1 male; 1 female	1 male; 3 female
Experience and training	Both Investigators received a PhD in epidemiology from an accredited school of public health. One investigator has previously conducted and published qualitative research studies	All GRAs were required to read a training manual on qualitative research procedures. All GRAs had training in human subjects research
Relationship with participants		
Relationship established	No relationship with focus group participants before study commencement	
Participant knowledge of the interviewer	Participants had no knowledge of the researcher’s personal goals or reasons for doing the research before focus groups were conducted.	
Interviewer characteristics	Participants were informed that the Investigators were researchers from local universities. GRAs were introduced as research assistants. Participants were told that the focus groups were being conducted as part of a National Institute of Justice funded study to create a screening tool for EMTs that would attenuate barriers to reporting elder abuse and neglect.	
Domain 2: Study design		
Theoretical framework	**EMTs**	**APS**
Methodological orientation and theory	Grounded Theory	
Participant selection		
Sampling	Participants were sampled conveniently	
Method of approach	All EMTs employed by the mobile healthcare provider and APS caseworkers were e-mailed by executive staff members at each agency (not the research team).	
Sample size	11	12
Non-participation	Executive staff members at the mobile healthcare provider and APS were responsible for recruiting participants. Given the sensitivity of this topic, the research team was not provided identifiable information about the participants (or potential participants) and information about non-participation could not be assessed.	
Setting		
Setting of data collection	Mobile healthcare provider office	Local APS branch office
Presence of non-participants	No persons other than the researchers and the participants were present during data collection	
Description of sample	Gender: 7 were men and 4 were women.	Gender: 11 were women, 1 man
	Race/Ethnicity: All were White, and one also identified as Hispanic.	Race/Ethnicity: One participant was White and the remainder were Black.
	Age: Mean was 40 years old (range 20-67)	Age: Mean of 39 years (range 23-63)
	Experience: Mean paramedic-level EMT for 7 years (range 2-22 years)	Experience: APS employee for 10 years (range <1-35)
Data collection		
Interview guide	The authors provided questions and prompts. However, the focus groups were semi-structured in nature and the conversation commonly deviated from the script.	
Repeat interviews	No repeat interviews were carried out.	
Audio/visual recording	Audio, but not visual, recording was used to collect data. After recording were transcribed by a GRA and verified by an Investigator, recordings were destroyed.	
Field notes	The secondary interviewer took field notes during each focus group.	
Duration	1 - 1.5 h	
Data saturation	The research team discussed data saturation after the first 3 focus groups and again after 2 additional focus groups. Data collection continued after the first 3 focus groups because the transcripts did not reflect saturation (new themes were being identified in focus group 3). After 5 focus groups, data collection was deemed complete, as no new themes were identified after transcript examination.	
Transcripts returned	Transcripts were not returned to participants for comments or corrections, as no identifiable information about participants was collected.	
Domain 3: Analysis and findings		
Data analysis		
Number of coders	Two coders coded data (one Investigator and one trained GRA)	
Description of the coding tree	There was no a priori coding tree created due to the limited theoretical knowledge base in this area. The two coders used a ‘two rivers’ approach to coding and identifying themes [33]	
Derivation of themes	Themes were derived from the data and not identified in advance	
Software	Dedoose 2.0 was used for data management	
Participant checking	Participants did not provide feedback on the findings. However, executive staff members at the mobile healthcare provider organization were provided a list of major themes.	
Reporting		
Quotations presented	Participant quotations are presented to illustrate themes.	
Data and findings consistent	There was consistency between the data presented and study findings.	
Clarity of major themes	All major themes relevant to the research question are discussed.	
Clarity of minor themes	Minor themes/diverse cases are discussed where relevant in the text.	

### Study population

At each site, senior administrators at each agency sent an e-mail to all employees (APS caseworkers and EMTs) with an invitation to participate in this study. The invitation made clear that participation was voluntary, and that choosing not to participate would not impact their employment. The senior administrators scheduled focus groups, and participants were paid by their agency as an incentive for participation. Agency administrators were not involved in data collection, and only research personnel not affiliated with either agency hosted, transcribed, coded and analyzed focus group data. Transcripts were not shared with each agency in light of the legal consequences associated with EMTs’ failure to generate a report to APS when elder abuse or neglect is suspected.

### Data collection

Five semi-structured focus groups ranging in size from 2 to 8 participants each, including 11 EMTs and 12 APS caseworkers (Total *N* = 23) were conducted. Although understanding barriers that EMTs face in reporting abuse was the focus of this study, APS was included in focus groups because they are charged with investigating any report of the abuse, neglect, or exploitation of an adult living with disability or an adult aged 65 or older [20]. If needed, APS provides services to the older adult and takes steps to prevent further harm [20]. Therefore, our understanding of barriers to abuse reporting would be impossible and largely incomplete without APS input.

EMT (*n* = 3) and APS (*n* = 2) focus groups were conducted on-site at each agency’s location between May and June, 2015. No administrators were present during data collection. When participants arrived for their scheduled focus groups, written informed consent was obtained, and participants completed a brief demographic questionnaire. A member of the research team informed participants that the purpose of these focus groups is to understand EMT experiences about elder abuse, barriers that might exist to reporting elder abuse, and identify methods for enhancing communication and reporting of potential elder abuse or neglect cases with APS. To minimize social desirability biases, a member of the research team instructed participants that there are no right or wrong answers, and that identifiable information will not be linked with their responses in any way.

Semi-structured focus groups lasted slightly longer than one hour (see Additional file 1 for interview guide). Focus groups began with a general discussion about case(s) of elder abuse encountered (for EMTs) or working with EMTs on a case that they reported (for APS) to encourage group discussions about field experiences [21]. EMTs were queried about why they did/did not report suspected cases of elder abuse, barriers to reporting, and whether they believe that it is “hard to determine” if elder abuse is occurring. At the end of each focus group, participants were asked to comment on how a hypothetical screening tool (if developed) might enhance: 1) the ease of reporting suspected cases of elder abuse or neglect by EMTs, 2) transmission of case information to APS, and 3) successful investigation.

All sessions were audio recorded and transcribed by a trained research assistant immediately after each focus group. To ensure anonymity, all participants were assigned a number that would be used in place of their name for the duration of the focus group. The Institutional Review Board at the University of North Texas Health Science Center approved the data collection protocol.

### Analytical methods

Systematic procedures of qualitative data analysis included: intensive reading of the text and group discussion of the transcripts by all members of the research team, coding by two investigators (a study co-Investigator and trained graduate student), inductive thematic identification, data reduction, and interpretation. These processes were iterative and coding occurred during the same time period for both coders (May-July, 2015). Inconsistencies in the coding process and findings were resolved by the research team. After the fifth focus group session, no new themes emerged in the transcripts (e.g., saturation was achieved) and no further focus groups were scheduled. All excerpts were coded by both coders without pre-determined themes in mind to minimize introduction of bias. Dedoose was used for all coding, organization and data reduction [22].

## Results

Demographic information about participants is provided in Table 1. Ninety-one percent of EMTs made at least one report of suspected elder abuse to APS during their lifetime. All EMTs expressed a desire to work more closely with APS and believed that they have a “*…responsibility [to report suspicions of elder abuse]. You take on a certain degree of responsibility if you don’t report it.”* When cases go un-reported, EMTs felt guilt, as illustrated by an EMT saying “*I actually should have [reported a case to APS] and I didn’t. I actually feel guilty.”*

The aim of the present study was to understand EMTs’ experience detecting and reporting elder abuse to APS. Findings suggest at least five barriers inhibited EMT’s ability to detect and/or report abuse or neglect. First, EMTs noted that older adults may elect or even prefer to live in environments that EMTs perceive as intrinsically neglectful. This reduces the EMT’s confidence in making the decision to report abuse, as “*[The older adult is] willingly living in a house with their daughter or niece, granddaughter, whoever, and it’s filth and you have roaches crawling allover the walls… and you sit there and [think], ‘is this really abuse?’”* In other words, EMTs perceive that the living conditions are normative to the older adult, or that the older adult might prefer to live in a neglectful lifestyle rather than being placed in a nursing home:

*“…They grew up in [that environment] and that’s what they’re used to, and that you know the 65-year-old, the woman … [who] was living in piles of trash, she wanted to live there and didn’t want to move out, she was fine living in that, and its not fair [to assume abuse or neglect] you know?”*

Similarly, one EMT reported:

*“APS has been out there and determined it was an [unsafe] situation and… removed the patient from the house and put them in a nursing home, and I have been out there for that… The patient is going ballistic all over them, ‘You can’t take me away from my house,’ and it’s just a sad case.”*

Second, the decision to report a suspected case to APS weighs heavily on EMTs, as they bear the moral burden of “wrecking someone’s life” based upon “gut” instincts that abuse may be occurring. EMTs also highlighted the consequences associated with reports of suspected elder abuse to APS. One EMT stated, “*How much do you want to invade their life, with getting the state involved, to maybe tear everything apart?”* EMTs were hesitant to judge older adult’s living conditions as abusive or neglectful given the consequences of reporting, but suggested that training or a checklist to guide their reporting decision would alleviate some of the emotional burden associated with reporting to APS.

Third, EMTs reported that time restrictions prevent them from reporting all cases of abuse that they encounter. For instance, because EMTs are dispatched immediately from one call to another, they have little time to locate a phone number for APS and transmit all of the details necessary to report a case. One EMT reported that a single phone report to APS “…*[has] taken me an hour to even get someone on the line…I had that time to actually sit. In the streets, you don’t have that time. So it is really frustrating,*” and this time commitment was corroborated by EMTs in each focus group. As a result, a situation “*has to be pretty outstanding for me [an EMT] to report it.*” Although an electronic reporting option is available, it is unreliable for EMTs, as they “*usually have to wait until after your shift [to contact APS], [be]cause the internet on the truck is spotty and it disconnects … so if I start a report and the network goes down, then everything I’ve done is deleted.”* In general, the current methods available to report suspicions of elder abuse and neglect to APS were repeatedly deemed as frustrating, time consuming, and burdensome. APS caseworkers also noted that telephone communication could be a barrier to reporting for EMTs, particularly in light of EMT’s time constraints. Because all calls to APS are routed through a central office (not local APS regional offices), systemic modifications to enhance communication between APS and EMTs are necessary.

Fourth, at the end of a 12-h shift, EMTs reported difficulty recalling sufficient information about a patient during a call to APS (“*It’s trying to remember enough, so when I call four hours later when I get off shift and get APS all the information they need”)* and,

*“Information is lost because in four hours, that’s four new patients, four new houses, four new calls”*

*“The past few nights we get back to back to back calls for the first 6-7 h of our shift, and then next five hours we’re not doing much, but how are you supposed to distinguish details between the first and last call?”*

As a result*, “you mix up information on a patient [from] another call,”* and data relayed to APS may not be accurate and result in an unfounded investigation. The fast-paced nature of the mobile healthcare industry requires user-friendly reporting protocols. When EMTs were asked if an automated reporting program, such as a checklist or screening tool would help them report cases, the response was overwhelmingly positive*: “If [reporting cases of suspected elder abuse to APS] were easier to do, I would report it every time I suspected it.”* Therefore, the data clearly suggest that the volume of patients seen by EMTs over the course of a single shift inhibits their ability contact APS and provide detailed information to file a report in a timely fashion. If new training or reporting programs were developed that could enhance EMTs’ ability to report suspected cases promptly and accurately, these more complete reports could lead to more successful APS investigations.

Finally, at the end of the focus groups, facilitators prompted EMTs to discuss the utility of a brief checklist or screening tool that could automatically generate and transmit a report to APS. Participants suggested that this type of instrument would increase their confidence in reporting potential cases of elder abuse or neglect to APS. EMTs noted that there is substantial “grey area,” (or uncertainty about reporting, the final barrier) and when they are fatigued after a long shift, subtle signals that could represent abuse or neglect might be overlooked. A checklist or screening tool would help ensure that EMTs were attentive to the circumstances that might warrant an APS report.

APS caseworkers sympathized with the barriers that prevent EMTs from reporting cases. In both APS focus groups, caseworkers noted that it is “*time consuming to submit a report either online or [via the hotline],”* and that “*EMTs just don’t have time [to report].”* Caseworkers agreed that calls to their hotline may last upwards of an hour, and that EMTs simply do not have the time to report all cases given their employment responsibilities.

In summary, EMTs consistently noted that, “*[contacting APS] was not an easy process… I had to bend backwards to do it.”* Notably, not a single EMT suggested that APS is easy to contact, or that reports can be generated in a timely manner. All EMTs agreed that they would “definitely” or “probably” use a screening tool if one were available, and EMTs agreed that training on the indicators of abuse and neglect is needed. These consistent findings highlight the need for an integrated reporting system that could automatically flag potential abusive or neglectful situations and generate a report transmitted to APS without addition burden on the EMT.

## Discussion

Findings from this study suggest that EMTs are eager to work with APS to address the under-reporting of elder abuse and neglect, but the current reporting procedures are time-prohibitive given their role as emergency healthcare providers. EMTs were largely supportive of new training programs or development of a brief checklist that would enable them to easily identify and communicate specific details about potential cases with APS during or immediately following calls for service while details are clear in their minds. Currently, EMTs receive only thirty minutes or less of training on elder abuse and neglect during their basic certification course. Therefore, additional continuing education courses should be focused on training EMTs to identify potential elder abuse or neglect and communicate the details of these cases with APS [23].

Almost all participating EMTs agreed that elder abuse is difficult to detect, and that there is “grey area” that may inhibit their ability to accurately detect situational and living conditions that may constitute abuse or neglect, particularly when they are fatigued at the end of their shifts. This finding is consistent with a previous qualitative study, which found that EMTs in Michigan reported only 27 % of suspected cases of abuse or neglect to authorities [23]. All EMTs agreed that a brief checklist would increase their confidence level in reporting to APS, and as a result, reduce their moral anxiety. These new protocols (e.g., training and checklists) could simplify and automate the processes associated with reporting suspected cases to APS, and as a result, link older adults in potentially dangerous situations with assistance.

Several screening tools for EA currently exist, but none to our knowledge are appropriate for use by EMTs in their current form [24–30]. For example, some existing tools require that questions be asked of the caregiver and/or the older adult [31]. If a caregiver is not present when EMTs enter a residence, these tools that require a caregiver response could not be completed. Other tools were designed for physicians and are simply not practical for prehospital care, field-based settings [24, 27]. In the field, EMT’s goal is to provide medical care; detection of elder abuse is ancillary. However, it is important to note that existing screening tools, such as the Elder Abuse Suspicion Index (EASI) [27], incorporate clinical judgement into the final assessment. Finally, other tools are very lengthy and cannot reasonably be completed in a field setting [32]. The limitations of previous developed screening tools highlight the need for a validated tool that relies upon EMT’s contextual observation rather than questionnaires.

### Strengths & limitations

Although results from this study were consistent across five focus groups, it is important to consider that data were collected from a small number of EMTs and APS caseworkers from North Texas. Future research on this topic should replicate these findings across other populations. Second, the long-term goal of this project was to develop a screening tool for EMTs, and this was briefly mentioned during informed consent ascertainment. Participants were told that the investigators seek to understand their unbiased opinions, but it is possible that the brief mention of the study purpose could have biased the discussion in favor of the screening tool. Participants were asked to provide very specific information about what factors would make them more or less likely to actually use a screening tool and many deterrents were identified (e.g., too many items, too many open-ended questions, questions are too long, etc.). Therefore, because focus group participants provided critical information (in addition to positive suggestions), we are confident that social desirability biases were minimized.

In light of these limitations, a number of strengths should be considered. First, this topic has great potential for large-scale public health impact if a method was developed to enhance the detection, reporting and successful investigation of elder abuse and neglect nationally. Second, the authors were able to gain stakeholder support from EMT and APS leadership, which allowed for this uncensored discussion of elder abuse and neglect reporting, a highly sensitive subject.

## Conclusion

Further developmental research is currently underway to identify which specific indicators of elder abuse and neglect identified from these focus groups are best suited for inclusion on a screening tool for EMTs. Using these indicators, randomized trials should be conducted to determine whether the screening tool truly enhances APS caseworkers’ ability to successfully investigate cases. Overall, results from the present study suggest that EMTs may be uniquely situated to serve as elder abuse and neglect surveillance personnel.

## Additional file

Additional file 1: Interview Guide. A semi-structured interview guide for APS and EMT focus groups. (DOCX 32 kb)

## Abbreviations

APS: Adult protective services
EA: Elder abuse, neglect, and exploitation
EMTs: Emergency medical technicians
FWSCC: Fort Worth [TX] Safe Communities Coalition
US: United States.
