# Supplementary material for: Barriers in detecting elder abuse among emergency medical technicians
Source: BMC Emerg Med. 2016 Sep 2;16(1):36. doi: 10.1186/s12873-016-0100-7 (PMC5010700; doi:10.1186/s12873-016-0100-7)
Supplement: Additional file 1: — Interview Guide. A semi-structured interview guide for APS and EMT focus groups. (DOCX 32 kb) [file 12873_2016_100_MOESM1_ESM.docx]

**Appendix A**

**Example Elder Abuse Screening Instrument Items**

|  | Directed Towards Caregiver |  |  |
| --- | --- | --- | --- |
|  | **Item** | **Response Options** | **Additional comments** |
| 1-12 | Caregiver:  Has behavior problem  Is financially dependent  Has mental / emotional difficulties  Has alcohol / substance abuse problem  Has unrealistic expectations  Lacks understanding of medical condition  Caregiving reluctancy  Has marital / family conflict  Has poor current relationship  Caregiving inexperience  Is a blamer  Had poor past relationship | 0 = nonexistent  1 = slight  2 = moderate  3 = probably / moderately severe  4 = yes / severe  00 = not applicable  000 = don’t know | (Reis & Nahmiash, 1998) |
| 13 | Is the client an older person who has a caregiver | Yes / No | (Reis & Nahmiash, 1998) |
| 14 | Is the client a caregiver of an older person | Yes / No | (Reis & Nahmiash, 1998) |
| 15 | Do you suspect abuse by a caregiver? | 1 = No, not at all  2 = Only slightly, doubtful  3 = Possibly, somewhat  4 = Probably, quite likely  5 = Yes, definitely | (Reis & Nahmiash, 1998) |
| 16 | Do you suspect abuse by a care receiver? | 1 = No, not at all  2 = Only slightly, doubtful  3 = Possibly, somewhat  4 = Probably, quite likely  5 = Yes, definitely | (Reis & Nahmiash, 1998) |

|  | Directed Towards Caregiver (continued) |  |  |
| --- | --- | --- | --- |
|  | **Item** | **Response Options** | **Additional comments** |
| 17 | What kind(s) of abuse(s) is (are) suspected? | Physical  Psychosocial  Financial  Neglect | (Reis & Nahmiash, 1998) |

|  | Directed Towards Older Adult |  |  |
| --- | --- | --- | --- |
|  | **Item** | **Response Options** | **Additional comments** |
| 1-15 | Care Receiver:  Has been abused in the past  Has marital / family conflict  Lacks understanding of medical condition  Is socially isolated  Lacks social support  Has behavioral problems  Is financially dependent  Has unrealistic expectations  Has alcohol / medication problem  Has poor current relationship  Has suspicious falls/injuries  Has mental / emotional difficulties  Is a blamer  Is emotionally dependent  No regular doctor | 0 = nonexistent  1 = slight  2 = moderate  3 = probably / moderately severe  4 = yes / severe  00 = not applicable  000 = don’t know | (Reis & Nahmiash, 1998) |
| 16 | Have you relied on people for any of the following: bathing, dressing, shopping, banking, or meals? | Yes / No / Did not answer | (Yaffe, Wolfson, Lithwick, & Weiss, 2008) |
| 17 | Has anyone prevented you from getting food, clothes, medication, glasses, hearing aides or medical care, or from being with people you wanted to be with? | Yes / No / Did not answer | (Yaffe et al., 2008) |
| 18 | Have you been upset because someone talked to you in a way that made you feel shamed or threatened? | Yes / No / Did not answer | (Yaffe et al., 2008) |
| 19 | Has anyone tried to force you to sign papers or to use your money against your will? | Yes / No / Did not answer | (Yaffe et al., 2008) |
| 20 | Has anyone made you afraid, touched you in ways that you did not want, or hurt you physically? | Yes / No / Did not answer | (Yaffe et al., 2008) |
| 21 | Can you take your medications and get around by yourself | Yes / No | (Neale, Hwalek, Scott, Sengstock, & Stahl, 1991) |
| 22 | Does someone in your family make you stay in bed or tell you you’re sick when you’re not? | Yes / No | (Neale et al., 1991) |
|  | Directed Towards Older Adult (continued) |  |  |
|  | **Item** | **Response Options** | **Additional comments** |
| 23 | Has anyone taken things that belonged to you without your OK? | Yes / No | (Neale et al., 1991) |
| 24 | Has anyone tried to hurt you or harm you recently? | Yes / No | (Neale et al., 1991) |

|  | Directed Towards a Third Party |  |  |
| --- | --- | --- | --- |
|  | **Item** | **Response Options** | **Additional comments** |
| 1 | Elder abuse may be associated with findings such as: poor eye contact, withdrawn nature, malnourishment, hygiene issues, cuts, bruises, inappropriate clothing, or medication compliance issues. Did you notice any of these today or in the last 12 months? | Yes / No / Did not answer | (Yaffe et al., 2008) |
| 2 | Mental Status | Alert / Confused / Unresponsive | (Fulmer & Cahill, 1984) |
| 3 | Hygiene | Yes / No | (Fulmer & Cahill, 1984) |
| 4 | Nutrition | Good / Fair / Poor | (Fulmer & Cahill, 1984) |
| 5 | Clothing | Good / Fair / Poor | (Fulmer & Cahill, 1984) |
| 6 | Maintenance of hygiene | Self / Assist | (Fulmer & Cahill, 1984) |
| 7 | Continent of bowel / bladder | Self / Assist | (Fulmer & Cahill, 1984) |
| 8 | Feedings | Self / Assist | (Fulmer & Cahill, 1984) |
| 9 | Ambulatory | Self / Assist | (Fulmer & Cahill, 1984) |
| 10 | If care provider present, is the observed relationship | Good / Poor / Indifferent / NA | (Fulmer & Cahill, 1984) |
| 11 | Physical Assessment (evidence of)  Bruising  Diarrhea  Dehydration  Lacerations  Urine Burns  Malnutrition  Abrasions  Decubiti  Alcohol Abuse | Yes / No | (Fulmer & Cahill, 1984) |
| 12 | Any duplication of similar medications? (i.e., multiple laxatives, sedatives, etc.) | Yes / No | (Fulmer & Cahill, 1984) |
| 13 | Any unusual doses of medications? | Yes / No | (Fulmer & Cahill, 1984) |
| 14 | Older adult has muscle contractures due to being restricted | Almost Always / Some of the Time / Never | (Ferguson & Beck, 1983) |
| 15 | Older adult is overly dependent on caregiver | Almost Always / Some of the Time / Never | (Ferguson & Beck, 1983) |

|  | Directed Towards a Third Party (continued) |  |  |
| --- | --- | --- | --- |
|  | **Item** | **Response Options** | **Additional comments** |
| 16 | Caregiver denies older adult’s illness | Almost Always / Some of the Time / Never | (Ferguson & Beck, 1983) |
| 17 | Caregiver shows evidence of loss of control, or fear of losing control | Almost Always / Some of the Time / Never | (Ferguson & Beck, 1983) |
| 18 | Caregiver presents contradictory history | Almost Always / Some of the Time / Never | (Ferguson & Beck, 1983) |
| 19 | Caregiver projects cause of injury onto third party | Almost Always / Some of the Time / Never | (Ferguson & Beck, 1983) |
| 20 | Caregiver has delayed unduly in bringing the older adult in for care, shows detachment | Almost Always / Some of the Time / Never | (Ferguson & Beck, 1983) |
| 21 | Caregiver overreacts or underreacts to the seriousness of the situation | Almost Always / Some of the Time / Never | (Ferguson & Beck, 1983) |
| 22 | Caregiver complains continuously about irrelevant problems unrelated to injury | Almost Always / Some of the Time / Never | (Ferguson & Beck, 1983) |
| 23 | Caregiver refuses to consent for further diagnostic studies | Almost Always / Some of the Time / Never | (Ferguson & Beck, 1983) |
| 24 | Caregiver is intrusive, allows older adult no privacy | Almost Always / Some of the Time / Never | (Ferguson & Beck, 1983) |

**References**

Ferguson, D., & Beck, C. (1983). HALF—A tool to assess elder abuse within the family. *Geriatric Nursing*, *4*(5), 301–304.

Fulmer, T. T., & Cahill, V. M. (1984). Assessing elder abuse: a study. *Journal of Gerontological Nursing*.

Neale, A. V., Hwalek, M. A., Scott, R. O., Sengstock, M. C., & Stahl, C. (1991). Validation of the Hwalek-Sengstock elder abuse screening test. *Journal of Applied Gerontology*, *10*(4), 406–418.

Reis, M., & Nahmiash, D. (1998). Validation of the indicators of abuse (IOA) screen. *The Gerontologist*, *38*(4), 471–480.

Yaffe, M. J., Wolfson, C., Lithwick, M., & Weiss, D. (2008). Development and validation of a tool to improve physician identification of elder abuse: the Elder Abuse Suspicion Index (EASI)\copyright. *Journal of Elder Abuse & Neglect*, *20*(3), 276–300.
